# Relapsed Acinic Cell Carcinoma of the Parotid Gland With Diffuse Distant Metastasis: Case Report With Literature Review

**DOI:** 10.1177/2324709616674742

**Published:** 2016-11-04

**Authors:** Yousef Khelfa, Munthir Mansour, Yousef Abdel-Aziz, Ali Raufi, Krista Denning, Yehuda Lebowicz

**Affiliations:** 1 Marshall University, Huntington, WV, USA

**Keywords:** salivary gland tumor, acinic cell carcinoma, parotid gland tumor

## Abstract

Acinic cell carcinoma (ACC) is an uncommon salivary gland neoplasm that generally displays an indolent growth pattern. Most cases arise in the major glands, particularly the parotid. However, it can arise from minor salivary glands in the oral cavity and aero-digestive tract. Although ACC is generally a low-grade malignant tumor, poorly differentiated and high-grade transformed variants exhibit a propensity for late recurrence and metastasis. There are no adequate clinical trials that define the optimal approach to patients with metastatic salivary gland tumors due to its rarity. Systemic therapy is reserved for cases where local therapy, such as radiation or metastasectomy, is not appropriate. Nevertheless, there is insufficient data in the literature regarding the chemotherapy of choice for metastatic ACC. In this article, we report a case of metastatic ACC of the right parotid gland that progressed on carboplatin and paclitaxel after partial response followed by doxorubicin and is currently on checkpoint inhibitor treatment.

## Introduction

Salivary gland tumors arise from either the major or the minor salivary glands and can be benign or malignant, with the parotid gland being the most common site, accounting for 85% of these tumors. Almost 25% of parotid gland tumors are malignant. The most common malignant salivary gland tumors include muco-epidermoid carcinoma and adenoid cystic carcinoma compromising together approximately one half of all malignant salivary gland tumors. Acinic cell carcinoma (ACC) is an infrequent malignant salivary gland tumor, and most cases arise in the major glands, particularly the parotid.^1-3^ ACC accounts for 6% of all primary salivary gland neoplasms and 17% of all primary malignant ones. Management of metastatic ACC is challenging due to limited data in the literature. We report a case of relapsed ACC of the right parotid gland with distant metastasis after initial improvement with neutron beam radiotherapy.

## Case Report

A 78-year-old female presented initially to our cancer center, after being diagnosed with ACC of the right parotid gland. She was complaining of a mass in the right side of her neck, which was associated with jaw discomfort. Her symptoms worsened gradually over 6 months. Head and neck computed tomography (CT) scan revealed soft tissue mass in the right side of the face, destroying the mandibular ramus and extending to the right sphenoid bone and inferior temporal lobe. A head and neck magnetic resonance imaging (MRI) scan showed a parotid tumor with extension into the foramen ovale and destruction of the right mandibular ramus. The patient underwent a fine needle aspiration biopsy, which showed ACC of salivary glands. The mass was deemed to be unresectable, and the patient was referred to radiation oncology department where she was treated with neutron beam radiotherapy. The parotid neoplasm responded well to treatment, and she had disease remission confirmed with follow up MRI.

A year later she developed right sided hip pain. After an unremarkable plain X-ray, she underwent a right femur and pelvis MRI that showed an osseous mass within the right ilium extending inferior to the acetabulum concerning for metastatic disease. CT of the abdomen and pelvis showed an abnormal lucency in the right acetabulum and a larger area of the right ilium, which measured 3.3 cm in maximum transverse dimension, as well as hepatic hypo-densities suggestive of metastatic disease. CT scan of the chest revealed numerous bilateral noncalcified lung nodules scattered throughout both lungs (Figure 2A) and a lytic bone lesion in the right scapula measuring 3.1 × 3.6 cm. This was followed by a bone scan that revealed uptake within the medial right ilium and the right inferior and superior pubic rami. There was also uptake within the right frontotemporal calvarium, right scapula, and anterior left third rib.

**Figure 1.**
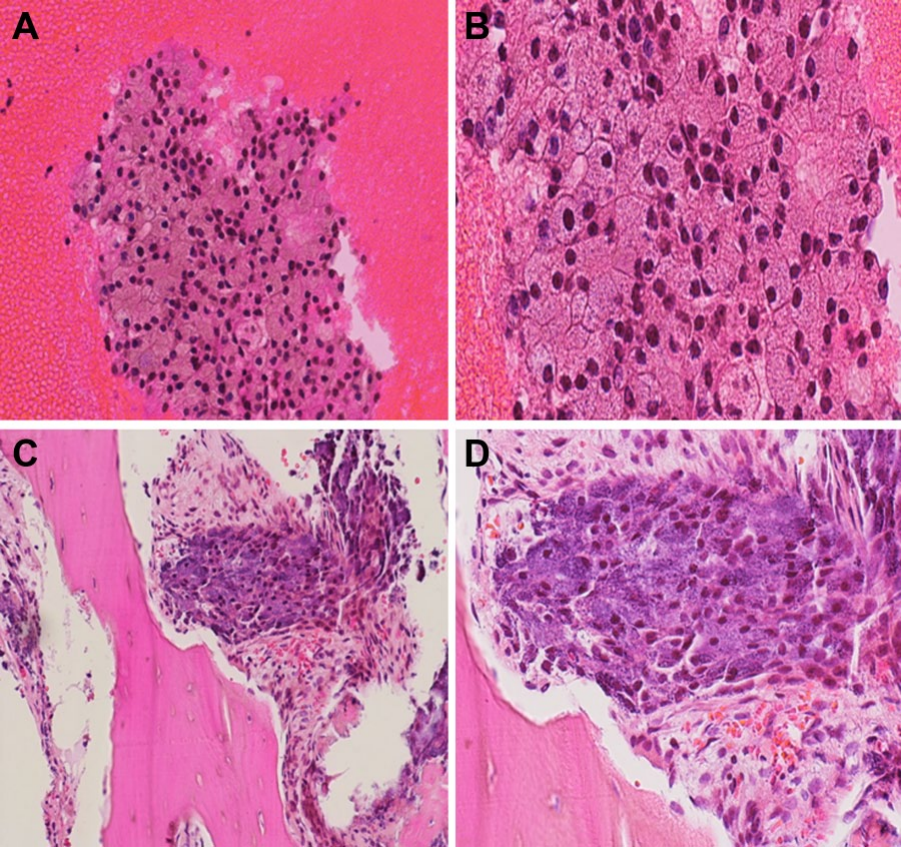
Cell block (high-magnification photomicrograph: A, 200×; B, 400×) of fine needle aspiration of the right ilium showing metastatic acinic cell carcinoma with serous acinar cells present in disordered microacinar arrangements. Hematoxylin-eosin (high-magnification photomicrograph: C, 200×; D, 400×) showing metastatic acinic cell carcinoma present in the right ilium bone with serous acinar cells with granular cytoplasm present.

**Figure 2.**
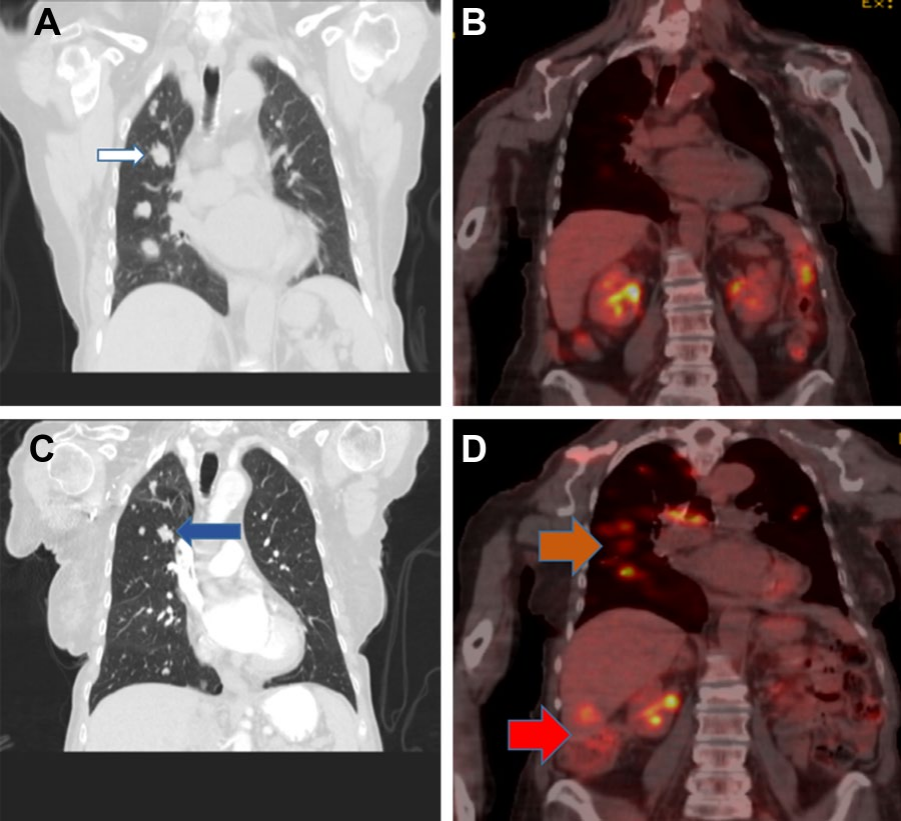
(A) CT chest shows pulmonary metastasis (white arrow). (B) PET-CT shows very good partial response. (C) CT chest shows pulmonary disease progression (blue arrow). (D) PET-CT scan shows pulmonary and hepatic disease progression (orange and red arrows, respectively).

Subsequently, she underwent right ilium biopsy, which confirmed the diagnosis of recurrent metastatic ACC (Figure 1). HER2 was checked by immunohistochemistry and fluorescent in situ hybridization and was negative. Next-generation sequencing (NGS) did not reveal targetable mutations. She was treated with 6 cycles of carboplatin and paclitaxel followed by single-agent paclitaxel. The patient tolerated the chemotherapy well, and a follow-up positron emission tomography-computed tomography (PET-CT) scan showed a significant partial response (Figure 2B). She remained progression free for 8 months when repeated CT scan showed disease progression (Figure 2C), and she was then treated with liposomal doxorubicin. Unfortunately, follow-up PET-CT showed disease progression again (Figure 2D). She has currently been on immunotherapy with PD1 inhibitor (pembrolizumab) for 6 months and has maintained an excellent quality of life.

**Figure 3.**
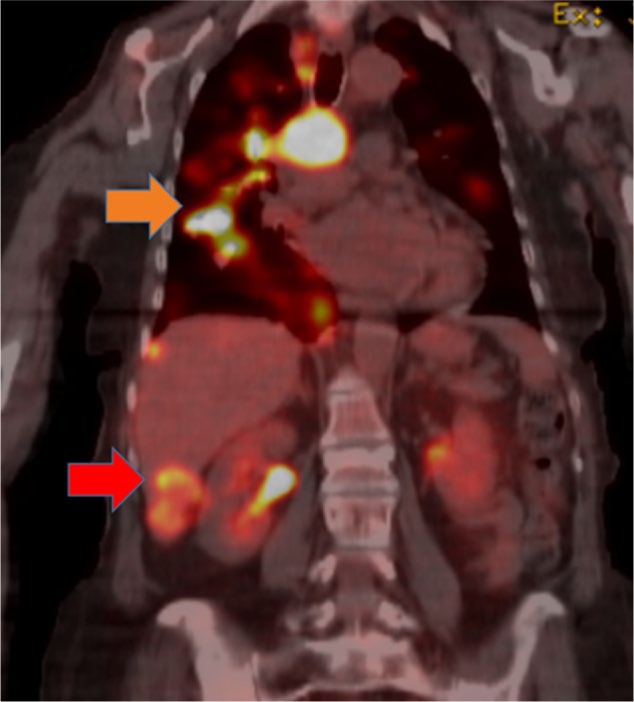
PET-CT scan shows both pulmonary and hepatic disease progression (orange and red arrows, respectively).

Despite improvement in her quality of life and excellent tolerance, PET-CT scan prior to the writing of this article showed further disease progression (Figure 3).

## Discussion

Salivary gland carcinomas are rare malignant tumors with diverse histopathology. It can be classified into high-grade tumors such as salivary duct carcinomas or low-grade tumors such as ACC. ACC is the third most common epithelial malignancy of the salivary gland. Most cases arise in the major glands, particularly the parotid. However, it can arise from minor salivary glands in the oral cavity and aero-digestive tract with the palate being the most common site. ACC accounts for 6% of all primary salivary gland neoplasms and 17% of all primary malignant salivary gland tumors. It is a relatively slowly growing tumor but has a propensity for late recurrence or metastasis, often years after initial presentation.^1,2,4,5^ Approximately 20% of patients will experience local recurrence and 10% will have distant metastasis, in contrast to high-grade tumors in which 33% will recur locally, and 46% will give rise to distant metastases.^1,2^ Minor salivary gland ACCs tend to have indolent behavior, with local recurrence being uncommon and distant metastasis very rare.^1^

Progression of disease is defined as local disease, regional spread, and distant metastasis. The survival rate is affected by the disease progression and histopathologic classification. Patel et al analyzed 1129 case of ACC between 1973 and 2009, and the results of this study were significant.^2^ Patients more than 60 years old accounted for 35% of cases and patients 40 to 59 years old accounted for 33%. Female-to-male ratio was 59.5% to 40.5%. Patients with localized disease had 5-, 10-, and 20-year survival rates of 100%, 99.15%, and 94.37%, respectively. On the other hand, patients with distant metastasis had 5-, 10-, and 20-year survival rates of 59.24%, 31.52%, and 21.99%, respectively. Patients with well-differentiated tumors had 20-year survival rates of 97.79%, and patients with moderately differentiated tumors had 20-year survival at 83.33%. However, patients with poorly differentiated ACC had 20-year survival rates of 38.06%.^2^

Histologically, ACC shows acinar differentiation, resembling serous cellular elements found in the normal parotid gland. Four architectural patterns have been recognized and include solid, papillary-cystic (macro-cystic), micro-cystic, and follicular. Different patterns may be recognized in a single lesion.1,6 The tumor cells are polygonal and lightly basophilic with granular cytoplasm. In addition to the serous cells, clear cells, intercalated duct cells, vacuolated cells, and nonspecific glandular cells may also be present.1 The cytoplasmic zymogen-like granules are key features in the diagnosis. Proteinaceous deposits similar to psammomatoid bodies and micro-calciﬁcations at various stages of development were infrequently reported.1 By immunohistochemistry the tumor cells are positive for cytokeratin and markers such as α-1 antichymotrypsin and 1-antitrypsin. Mammary analogue secretory carcinoma of salivary glands (MASC) may resemble ACC histologically. However, unlike ACC there are no true acinar components in MASC. MASC is often strongly S100 positive and their deﬁning feature is the presence of t(12; 15) (p13; q25) translocation that results in ETV6-NRTK3 gene fusion.4,5,7 ACC can occasionally undergo a high-grade transformation into an adenocarcinoma or undifferentiated carcinoma. Usually, the salivary neoplasm becomes highly aggressive and the patient occasionally presents with metastatic disease. Warner et al reported a case of aggressive metastatic ACC; NGS analysis reported deletions in tumor suppressor genes CDKN2A (cyclin dependent kinase inhibitor 2A) and CKDN2B and non-sense mutation in ARID2.6 CDKN2A encodes P16INK4A and P14ARF. CDKN2B encodes P15INK4B. P16INK4A and P15INK4B induce cell cycle arrest by complexing to CDK4 and CDK6 (cyclin dependent kinase). They prevent phosphorylation of retinoblastoma protein, which inhibits cell cycle progression. The Food and Drug Administration approved CDK4/6 inhibitor palbociclib for treatment of metastatic breast cancer. Since these mutations are identified in certain patients with metastatic ACC, the benefit of CKD4/6 inhibitors should be studied.6 In fact, our patient has a mutation in CDKN2A/B.

Clinically, patients with a tumor of the major salivary gland typically present with a painless mass or swelling of the salivary glands. The presence of a parotid mass in combination with facial nerve paralysis is generally indicative of a malignant rather than a benign tumor. Minor salivary gland tumors arising within the oral cavity may present with a painless submucosal mass or mucosal ulceration. Lymphatic drainage varies according to location of the salivary gland. In parotid malignancies, the first site of lymphatic spread is the intraparotid lymph nodes, followed by level I and level II cervical nodes. Submandibular gland tumors spread to adjacent perivascular nodes and then to the cervical region. The sublingual gland drains to the submental and submandibular nodes, and the minor salivary glands drain to the retropharyngeal nodes. Although ACC tends to have an indolent course, distant metastasis can still occur. The most common sites reported for distant metastasis include the lungs, bone, and the liver.^1,2,5^ Patients with ACC may have increased risk for other malignancies. Omlie and Koutlas reported 21 cases of ACC, one patient developed non-Hodgkin lymphoma and another was found to have renal cell carcinoma.^1^

Management of metastatic ACC is very challenging. Because of the rarity of these cases, data on management are based mainly on clinical experience and retrospective series. Treatment of local disease remains preferably surgical resection. Radiotherapy, particularly fast neutron-beam radiation therapy, has been advocated for use either instead of surgery or postoperatively in cases of positive margins after resection, extra-capsular extension, nodal involvement, presence of tumor necrosis, inoperable tumor, and recurrent disease.^4,8,9^ North et al concluded that radiotherapy is recommended postoperatively for all cases of salivary gland cancer except for those tumors staged as T1N0 or T2N0 with low-grade histology, which were excised with negative margins.^10^ In case of local relapse, attempts should be made for complete surgical resection, since this provides the best chance for potential disease cure.^10^ If complete and total tumor removal is not achievable, radiation should be considered. In cases of metastatic ACC, curative surgical resection of a solitary site of metastasis should be considered. Concurrent chemo-radiotherapy seems to be promising as reported by Tanvetyanon et al.^11^ The results of their case-control study showed increased overall survival in the group given chemo-radiation with a platinum based regimen versus the radiation alone group.

There is no clear evidence that survival is prolonged by systemic therapy.^12^ Thus, systemic therapy is most often palliative and is reserved for those for whom local therapy, such as radiation or metastasectomy, is not appropriate.^12^ There is insufficient data in the literature regarding the chemotherapy of choice for metastatic ACC.^2,9,12^ Most of the studies included adenoid cystic carcinoma, adenocarcinoma, and muco-epidermoid carcinoma, but data on metastatic ACC are scarce. It was suggested that the sensitivity to different chemotherapy regimens varies depending on the histologic subtype of salivary gland tumors.^12^ Platinum-based chemotherapy as monotherapy or as a combined regimen remains the most common used regimen for treatment of metastatic salivary gland tumors. Although studies showed higher objective response rate with combined regimens, they failed to demonstrate any actual increased survival rate. Cyclophosphamide, doxorubicin, and cisplatin (CAP) is the most common studied regimen with an overall response rate of 46%.^9,12,13^ Other regimens include PAF (cisplatin, doxorubicin, and 5-flourouracil) and CAPF (cisplatin, doxorubicin, cyclophosphamide, and 5-flourouracil), which was associated with response rate of 50% but with higher chemotherapy-related side effects.^9,12,14^ Vidyadhara et al reported a patient with metastatic ACC of parotid gland who was treated with 9 cycles of cisplatin, epirubicin, and 5-flourouracil. He experienced improvement in his symptoms initially but his disease progressed after 6 months.^15^

The role of molecular targeted therapy for treatment of metastatic ACC is not well established. This is partially because there are no major translocations and fusion oncogenes identified in ACC. Several biologic therapies have been used and reported as retrospective series with variable results in other salivary neoplasm subtypes. Trastuzumab showed promising results in some muco-epidermoid carcinomas and salivary duct carcinomas. Studies using imatinib, dasatinib, cetuximab, sorafenib, and bortezomib did not show significant objective response. Disease stability was observed in a few cases; however, the significance of this observation is questionable considering the fact that salivary gland tumors are in general slow-growing tumors. A preliminary result of the phase 1b KEYNOTE-028 was presented at the annual 2016 ASCO meeting, in which 26 heavily pretreated patients with advanced (unresectable and/or metastatic) salivary gland carcinoma received pembrolizumab 10 mg/kg every 2 weeks for up to 24 months or until confirmed progression, intolerable toxicity, death, or withdrawal of consent. Pembrolizumab showed promising antitumor activity.^16^ Clinical benefit of pembrolizumab in advanced salivary gland carcinoma will be further investigated in the phase 2 KEYNOTE-158 trial.^17^ Our patient showed significant partial response to carboplatin and paclitaxel. She remained progression free for 8 months. On progression she was treated with liposomal doxorubicin; however, disease unfortunately progressed while on therapy. She has subsequently been on immunotherapy with PD1 inhibitor (pembrolizumab), and after a dramatic clinical improvement, she has maintained an excellent quality of life for 6 months.

Unfortunately, she developed a solitary brain metastasis very near the prior field of radiation.

Repeat systemic imaging has now showed widespread progression.

She is currently undergoing radiosurgery to central nervous system metastasis. We are discussing consideration of palbociclib versus hospice care. Due to the decline in performance status secondary to brain metastasis she is leaning toward comfort measures alone.

## Conclusion

Acinic cell carcinoma is an infrequent malignant salivary gland tumor. Most cases arise in the major glands, particularly the parotid. However, ACC can arise from minor salivary glands, are usually asymptomatic and slow growing, and treated primarily with local excision. There are no adequate clinical trials that define the optimal approach to patients with metastatic salivary gland tumors because of the rarity of these tumors. Chemotherapy is reserved for systemic disease when local therapy is not appropriate. The primary goal of chemotherapy is palliation of disease-related symptoms and perhaps improvement in Progression Free Survival (PFS)and Overall Survival (OS). We report a case of relapsed metastatic ACC of the right parotid that showed significant response and progression-free survival for 8 months with systemic carboplatin and paclitaxel. Whether checkpoint inhibitors will play a major role in treatment of this cancer is a question yet to be fully answered by ongoing clinical trials. Our patient progressed on this line of treatment; however, her quality of life had dramatically improved while on therapy. Further research into both targeted and immunotherapy is eagerly awaited and needed.
